# Nutrition and Fitness: Mental Health

**DOI:** 10.3390/nu12061804

**Published:** 2020-06-17

**Authors:** Riccardo Dalle Grave

**Affiliations:** Department of Eating and Weight Disorders, Villa Garda Hospital, Via Monte Baldo, 8937016 Garda (VR), Italy; rdalleg@gmail.com

Mental disorders are one of the leading causes of disability, being associated with about 18.9% of years lived with a disability [1]. Traditionally, depressive disorders, bipolar disorders, schizophrenia, anxiety disorders, and eating disorders have been treated with psychopharmacological drugs, including antipsychotics, antidepressants, mood stabilizers, and antianxiety agents, and/or different forms of psychotherapies. Unfortunately, with these treatments, the burden of mental disorders is prevented in less than 50% of cases, an outcome indicating the need to find new additional strategies and procedures to improve their management [2].

*Mens sana in corpore sano* (a healthy mind in a healthy body) is a Latin phrase taken from Giovenale (Satire, X, 356) that remains relevant and is supported by today’s data regarding nutrition and physical activity, and their contribution to mental health. Indeed, several data have found an association between nutrition physical fitness and mental health [2,3,4,5], supporting the potential role of using nutrients and physical activity as agents for prevention, treatment, or augmentation of treatment for mental disorders in children, adolescents, and adults.

The Special Issue “Nutrition and Fitness: Mental Health” of *Nutrients* includes four original articles [6,7,8,9] and three systematic reviews [10,11,12]. The first original article assessed the interconnections between specific quality of life domains (assessed with Short Form-36 [SF-36]) in 716 consecutive female and male patients with obesity and high or low physical performance using an innovative statistical analysis based on network approach [6]. Low-performing patients (64.7% of the sample) reported lower quality of life domain scores, but the network structures were similar in the two groups, with the SF-36 Vitality representing the central domain in both networks. Moreover, in patients with obesity and low physical performance levels, mental health was a central variable, indicating that psychological aspects should be considered in defining the quality of life in patients with low physical performance levels.

In the second original article, 162 individuals who were obese or overweight were randomly allocated to four interventions, 24 weeks in duration [8]: strength, endurance, combined strength + endurance, and guideline-based physical activity; all in combination with a 25–30% caloric restriction diet. The study found several positive effects of the intervention on energy intake, macronutrient selection, and body composition changes, with a significant reduction of body mass index and body fat percentage, but no significant differences of exercise type. The study also confirmed that individuals allocated to a long-term exercise program associated with dietary advice do not increase their energy intake in a compensatory fashion.

The third original article randomly allocated 51 patients with multiple sclerosis to 800 mg of epigallocatechin gallate (EGCG) and 60 mL of coconut oil or placebo for four months [7]. Both groups followed the same isocaloric Mediterranean diet. EGCG and coconut oil decreased state anxiety and functional capacity, while the levels of interleukin 6 (IL-6) decreased in both groups, likely because of the antioxidant effect of the Mediterranean diet.

The fourth original article investigated the public health topic of the association between food insecurity (i.e., the presence of limited or uncertain availability or access to nutritionally sufficient, socially relevant, and safe foods) and depressive symptoms in 8613 adults who participated in the Indonesia Family Life Survey (IFLS) in 2007 and 2014 [9]. The study found a positive association between depressive symptoms and food insecurity: a finding that underlines the importance to implement specific nutritional and health programs to prevent and treat both food insecurity and mental health.

The first systematic reviews synthesized data from 14 studies to assess and discuss the potential role of microbiota-orientated treatments (including fecal microbiota transplantation (FMT) in major depression and schizophrenia) [10]. The results indicate that probiotics seem to have a medium-to-large significant effect on depressive symptoms, but it is not clear if these positive effects are maintained after probiotic discontinuation. Since FTM has been shown to improve microbiota in several gut disorders, the authors suggest that this procedure may be a potential strategy to test for improving the efficacy of microbiota-orientated treatments in major depression and schizophrenia and maintain their effect over time.

In the second systematic review, the authors synthesized the data of 25 studies (ten experimental and 15 observational studies) assessing the relationship between energy balance-related behavior (i.e., physical activity, sedentary, and dietary behavior) and burn-out risk [11]. Physical activity seems effective in reducing burn-out, as supported by the data of nine experimental and 14 studies. On the contrary, although the data of few observational studies suggest that being sedentary and eating less healthily are both associated with higher burn-out risk, there is a need for more high-quality research to reach meaningful conclusions on this association.

However, when the physical activity becomes excessive and compulsive, a distinctive behavioral feature of a subgroup of patients with eating disorders [13], it is associated with more severe general and eating disorder psychopathology, as synthesized by the third systematic review [12] of this Special Issue. The authors, who analyzed 47 articles, suggest using the term “problematic use of physical activity (PPA)” to define this unhealthy form of exercising and propose an original model for the development of PPA in patients with anorexia nervosa, encompassing five periods evolving into three clinical stages. They also suggest the presence of two components of PPA in anorexia nervosa: (i) voluntary PPA to influence body shape and weight; and (ii) involuntary PPA that it is biology driven and increases with weight-loss. Future research will have to test the theory proposed by the Authors and its clinical utility.

In conclusion, the findings of the original articles and systematic reviews of this Special Issue confirm that nutrition and physical activity seem to play an important role in maintaining good mental health and are two potential interventions to improve the management of mental disorders.
